# Positional Bias of MHC Class I Restricted T-Cell Epitopes in Viral Antigens Is Likely due to a Bias in Conservation

**DOI:** 10.1371/journal.pcbi.1002884

**Published:** 2013-01-24

**Authors:** Yohan Kim, Jonathan W. Yewdell, Alessandro Sette, Bjoern Peters

**Affiliations:** 1 La Jolla Institute for Allergy & Immunology, La Jolla, California, United States of America; 2 Laboratory of Viral Diseases, National Institute of Allergy and Infectious Diseases, Bethesda, Maryland, United States of America; Emory University, United States of America

## Abstract

The immune system rapidly responds to intracellular infections by detecting MHC class I restricted T-cell epitopes presented on infected cells. It was originally thought that viral peptides are liberated during constitutive protein turnover, but this conflicts with the observation that viral epitopes are detected within minutes of their synthesis even when their source proteins exhibit half-lives of days. The DRiPs hypothesis proposes that epitopes derive from **D**efective **Ri**bosomal **P**roducts (DRiPs), rather than degradation of mature protein products. One potential source of DRiPs is premature translation termination. If this is a major source of DRiPs, this should be reflected in positional bias towards the N-terminus. By contrast, if downstream initiation is a major source of DRiPs, there should be positional bias towards the C-terminus. Here, we systematically assessed positional bias of epitopes in viral antigens, exploiting the large set of data available in the Immune Epitope Database and Analysis Resource. We show a statistically significant degree of positional skewing among epitopes; epitopes from both ends of antigens tend to be under-represented. Centric-skewing correlates with a bias towards class I binding peptides being over-represented in the middle, in parallel with a higher degree of evolutionary conservation.

## Author Summary

To defend the host from an infection, the immune system continuously scans cell surfaces for foreign objects. Specifically, a virus inside a cell exploits the host to make copies of its proteins; viral proteins are broken up into peptide fragments; and the fragments are displayed on the infected cell's surface, thereby allowing detection and cell-killing. How these peptide fragments for cell-surface presentation are generated remains unknown. An understanding of this step will lead to rational design of vaccines and insights into tumor immunosurveillance and autoimmunity. One possible mechanism is that the peptide fragments come from defective proteins missing either the beginning or end regions, which may result in a bias. Here, we analyzed locations of a large set of known viral epitopes, peptide fragments recognized by the immune system, within their proteins. We find that all regions of proteins are represented well by the immune system. However, there is a statistically significant bias in the central regions of proteins, which correlate with a pattern of conservation spanning the length of viral proteins. Our results suggest a combined effect of conservation and enhancement of immune responses through repeated exposures in shaping the distribution of known viral epitopes.

## Introduction

The immune system rapidly detects virus-infected cells through cell-surface presentation of viral peptides to T-cells despite the fact that the half-lives of source proteins are typically orders of magnitude longer than the response time (i.e. days vs. minutes) [1], [2]. To explain this paradox, the DRiP hypothesis proposed that *defective ribosomal products*, rapidly degraded forms of standard gene products, are a major source of peptides for MHC class I processing pathway [3].

Although the DRiPs hypothesis is well into its teens, surprisingly little is known about the nature of the DRiPs [4]–[6]. This is likely due to the low abundance of DRiPs relative to the abundance of folded proteins, which poses a significant challenge to current biochemical/molecular technologies. A central question is what mechanisms dominate in the production of DRiPs [5], [6]. One set of mechanisms that leads to the generation of partial protein products is downstream initiation and premature terminations when translating proteins from mRNAs [7]. If such errors are involved in generation of DRiPs, a bias in sampling of regions of mRNAs may result. This sampling bias in turn may influence from which regions of proteins epitopes are more often detected, resulting in *positional bias of epitopes*. For example, a dominance of downstream initiation would result in more epitopes detected from the C-terminal ends; conversely, a dominance of premature termination would result in more epitopes detected from the N-terminal regions.

To overcome the limitation of the current experimental approaches, we have investigated if a data-driven approach can provide insights into the nature of DRiPs. Namely, the availability of a large repository of immune epitopes stored at the Immune Epitope Database (IEDB) [8] makes it possible to indirectly characterize the population of DRiPs by measuring positional bias of viral epitopes.

## Results

### A summary of MHC class I restricted viral T-cell epitopes available in the IEDB

Because viruses exploit the translational machinery of the host to synthesize their proteins and are thus relevant in the context of the DRiPs hypothesis, we retrieved all MHC-I restricted T-cell epitopes of viruses for which reference proteomes are available. Top 20 viruses based on number of tested peptides are shown in **Table 1**. For each virus, total number of unique peptides tested, peptides with positive T-cell outcomes (i.e. epitopes), and those with negative outcomes are shown. In the table, *vaccinia virus* contributed the greatest number of tested peptides, followed by *hepatitis C Virus*, *lymphocytic choriomeningitis virus*, *human herpesvirus 5/4* and *influenza A virus*.

**Table 1:** MHC class I restricted T-cell epitopes retrieved from the Immune Epitope Database for viral species.

Organism Name	All	Positive	Negative
Vaccinia virus	8612	414	8198
Hepatitis C virus	1637	622	1015
Lymphocytic choriomeningitis virus	1046	100	946
Human herpesvirus 5	997	433	564
Human herpesvirus 4	839	255	584
Influenza A virus	655	303	352
Murine coronavirus	468	22	446
Dengue virus	204	103	101
Human respiratory syncytial virus	187	32	155
Yellow fever virus	187	49	138
Murid herpesvirus 1	184	42	142
Hepatitis B virus	179	88	91
Equine infectious anemia virus	155	90	65
Primate T-lymphotropic virus 1	151	124	27
Human papillomavirus - 16	130	76	54
Hantaan virus	122	12	110
Human herpesvirus 8	99	78	21
Human herpesvirus 1	98	80	18
Theilovirus	97	16	81
West Nile virus	82	59	23

For each organism, total number of tested peptides as well as numbers of those with positive and negative assay outcomes are shown. A total of 93 viruses were studied. For brevity, 20 viruses with the highest number of tested peptides are shown in the table.

### Positional bias of known MHC class I restricted T-cell epitopes from viruses

**Figure 1:**
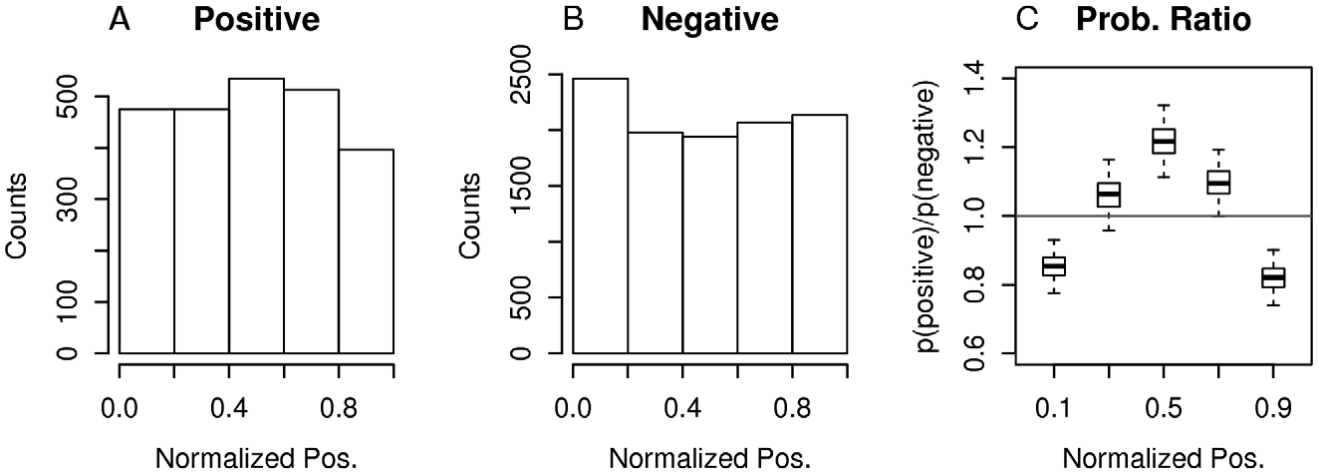
Distributions of normalized positions of MHC-I restricted T-cell epitopes for viruses. (A and B) For each category of peptides indicated (i.e. ‘Positive’ or ‘Negative’), peptides were mapped onto all proteins of the corresponding genome with peptide similarity cutoff of 100%. For each peptide:antigen mapping, a normalized position was calculated. The set of normalized positions was plotted as a histogram. (C) To show positional bias of epitopes, the box plot shows results of 1000 bootstrap sampling of Positive and Negative data sets of normalized positions and plotting ratios of their probabilities for each bin. Boxes cover the range from 25^th^ to 75^th^ percentiles. Whiskers extend out from boxes 1.0 times the interquartile range.

After determining positions of viral peptides in their reference antigens and calculating normalized positions, we constructed distributions of normalized positions for positives (i.e. epitopes) and negatives as shown in **Fig. 1A** and **1B**, respectively. Each distribution used 5 bins of equal intervals covering a range [0,1]. Distributions plotted using 10 bins showed similar patterns (data not shown). The distributions correspond to probability mass functions, p(x|positive) and p(x|negative), where ‘x’ represents a binned normalized position (described further in Methods section).

To show positional bias of epitopes, a probability ratio plot (i.e. p(x|positive)/p(x|negative), see Methods for details) is shown in **Fig. 1C**. By dividing the distribution for positive peptides by the distribution of negative peptides, we implicitly take into account study biases that are otherwise difficult to capture. If positional bias is absent, the corresponding probability ratio plot would show a horizontal line at a probability ratio of 1. If positional bias is present, regions with lower likelihoods of finding epitopes would show values less than 1. Indeed, in **Fig. 1C**, the probability ratio plot shows under representation of epitopes at N- and C-termini. Furthermore, probability ratio plots generated after cumulative exclusion of data from vaccinia virus and Hepatitis C virus, which contributed most in terms of data, resulted in maintaining the overall pattern (supplementary Fig. S1)

The positional bias of epitopes observed is supported by results of statistical analyses on the corresponding contingency table (supplementary **Table S1**). Using a binomial test for each bin, it was determined whether counts of positives significantly deviate from the expected fraction of positives (i.e. (all positives)/(all positives+all negatives) = 2394/12974). Out of 5 bins, 4 deviated from the expected (two sided; p-value<0.05).

### Absence of positional bias of predicted MHC class I binding peptides in viral antigens

A possible source of positional bias of epitopes is unequal distributions of amino acids spanning the length of antigens. For instance, the positional bias observed may have been due to hydrophobic residues being preferentially found in middle regions rather than at N- and C-termini. Such unevenness would mean that MHC alleles binding peptides with hydrophobic anchor residues would impose positional bias of epitopes. To rule out this possibility, we determined positional bias of *predicted binding peptides* of viruses to MHC molecules.

Our prediction strategy uses recently developed peptide:MHC-I binding algorithms, which have achieved high accuracies in benchmarks [9], [10]. Using 9-mer peptides generated from a set viral proteins, we made binding predictions to human MHC-I molecules (HLA). As a note, HLA molecules can be grouped into 12 HLA supertypes based on their known overlap of binding specificities [11]. SMM^PMBEC^ was used to make binding predictions [12]. Corresponding member alleles for each supertype used for predictions are provided in the supplementary material (**Table S2**). After grouping predictions based on supertypes, for each supertype, we generated distributions of normalized positions for predicted ‘binders’ (i.e. those peptides with predicted binding affinities <500 nM) and ‘non-binders’ (i.e. those with affinities >500 nM).

**Figure 2:**
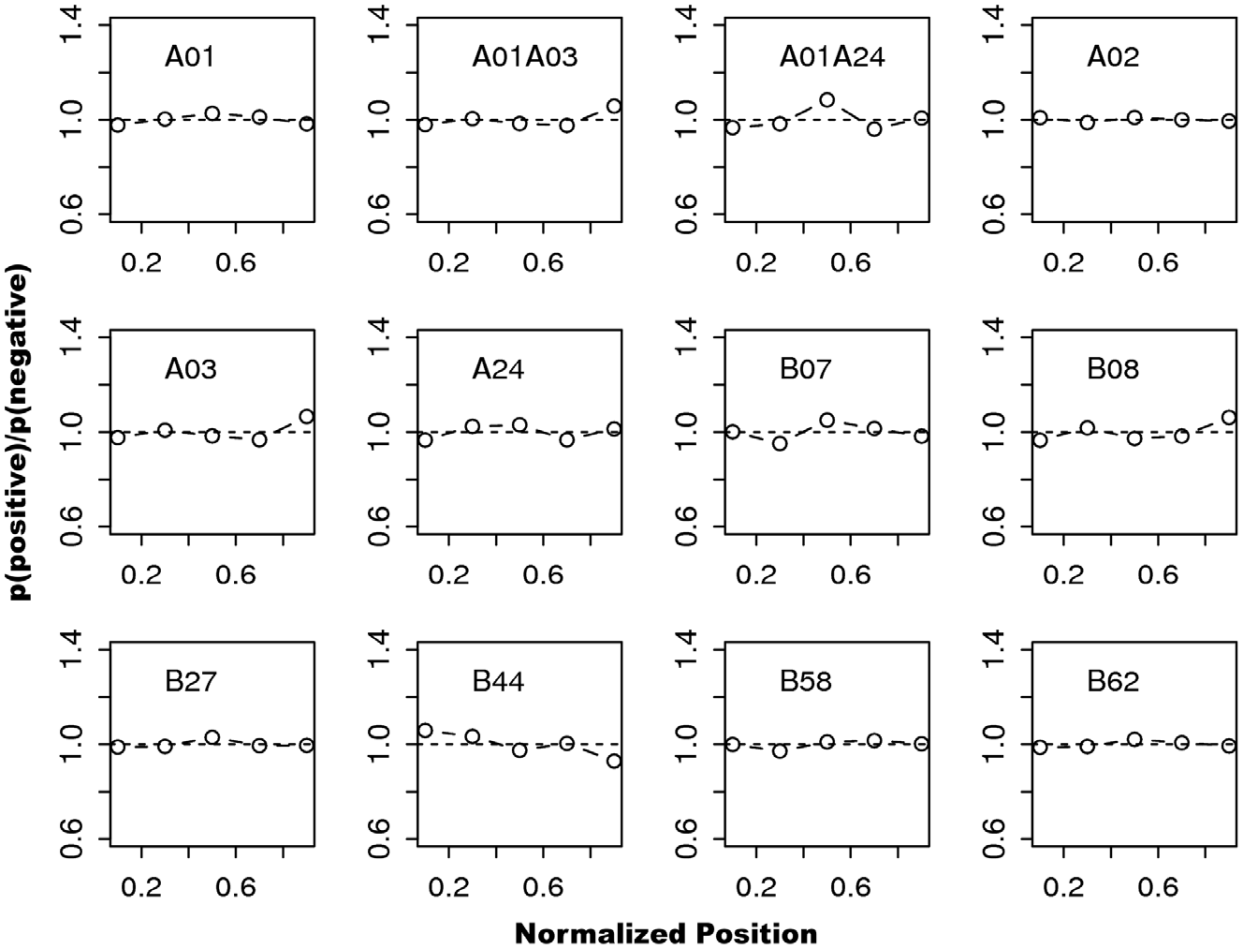
Positional biases of predicted binders for 12 HLA supertypes. For each supertype, 9-mer peptide binding predictions were carried out and ratios of probability masses of predicted ‘binders’ and ‘non-binders’ were calculated. Peptide binding predictions were made for alleles belonging to each supertype, using SMM^PMBEC^ method. All possible 9-mer peptides were generated from a set of viral proteins that contain at least one tested peptide from Table 1. Relationships between HLA molecules and supertypes are provided in [11].

Probability ratio plots derived from the distributions for 12 HLA supertypes are shown in **Fig. 2**. The lack of divergence from a horizontal line at 1.0 indicates absence of positional bias. In the figure, some of the supertypes show slight positional bias. Specifically, supertype A01 favors the middle region of antigens, whereas A03 favors the C-terminus. A combined plot generated from weighted averaging of probability ratio plots from all supertypes based on frequencies of known MHC restriction data showed absence of positional bias (supplementary Fig. S2). To ensure that these observations are not due to the choice of predictive method, we used a different predictive method NetMHC [13], which is another accurate predictive method, and made the same observations. In addition, scatter plots of predicted binding affinities for the tested peptides and their normalized positions showed no systematic effects (supplementary Fig. S3).

The presented results largely reflect results reported in [14], where mammalian, bacterial, and viral proteomes show lack of influence of MHC class I binding preferences. However, in the same work, A02 and B07 supertypes showed bias in signal-peptide regions (i.e. N-terminal). We confirmed that using our prediction strategy, signal-peptide specific biases observed for A02 and B07 are present for both human and viral antigens if *residue positions* are used instead of normalized positions (data not shown).

### Positional bias of protein conservation correlates with positional bias of viral epitopes

A DRiP-independent factor that could explain the positional bias of viral epitopes is positional bias of protein conservation. Specifically, if ends of proteins are less conserved than the middle region and epitopes tend to be more conserved than non-epitopes, positional bias of epitopes may result. To test this possibility, we calculated conservation scores at the residue-level for proteins of the viruses (See Methods for details on calculation of conservation scores). Conservation scores could be assigned to ∼60% of proteins. The remaining proteins had an insufficient number of suitably distant homologues to construct reliable conservation scores, and were therefore excluded from this analysis.

**Figure 3:**
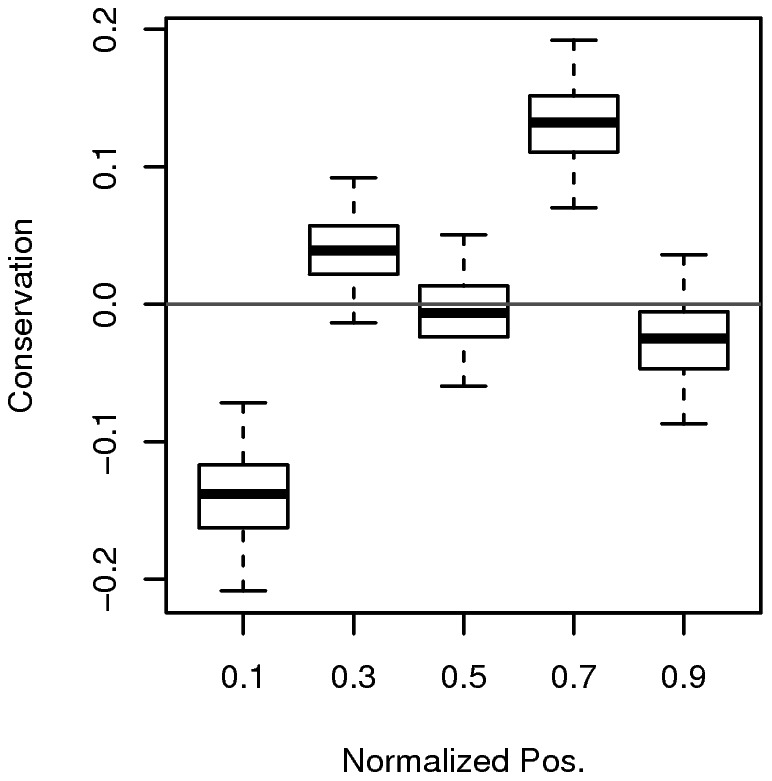
Positional bias of conservation for viral proteins. For each viral protein which also contained at least one tested peptide from Table 1, conservation scores at the residue-level were calculated using Rate4Site [26]. Residue positions were converted into normalized positions and corresponding conservation scores were binned (5 bins of equal size). Higher conservation score indicates higher degree of conservation. Conservation scores were normalized for each protein (i.e. mean = 0; sd = 1). One thousand bootstrapping over proteins was used to estimate confidence intervals for the means of conservation scores. Each box covers 25^th^ and 75^th^ percentiles. Whiskers extend out from each box 1.0 times the interquartile range.

In **Fig. 3**, a boxplot showing confidence intervals of means of conservation scores as a function of normalized position is shown. This reveals a pattern of positional bias of conservation, where ends of proteins are less conserved than their cores, similar to the pattern observed for the positional bias of known epitopes in **Fig. 1C**. For the middle bin, however, we do see a difference.

**Figure 4:**
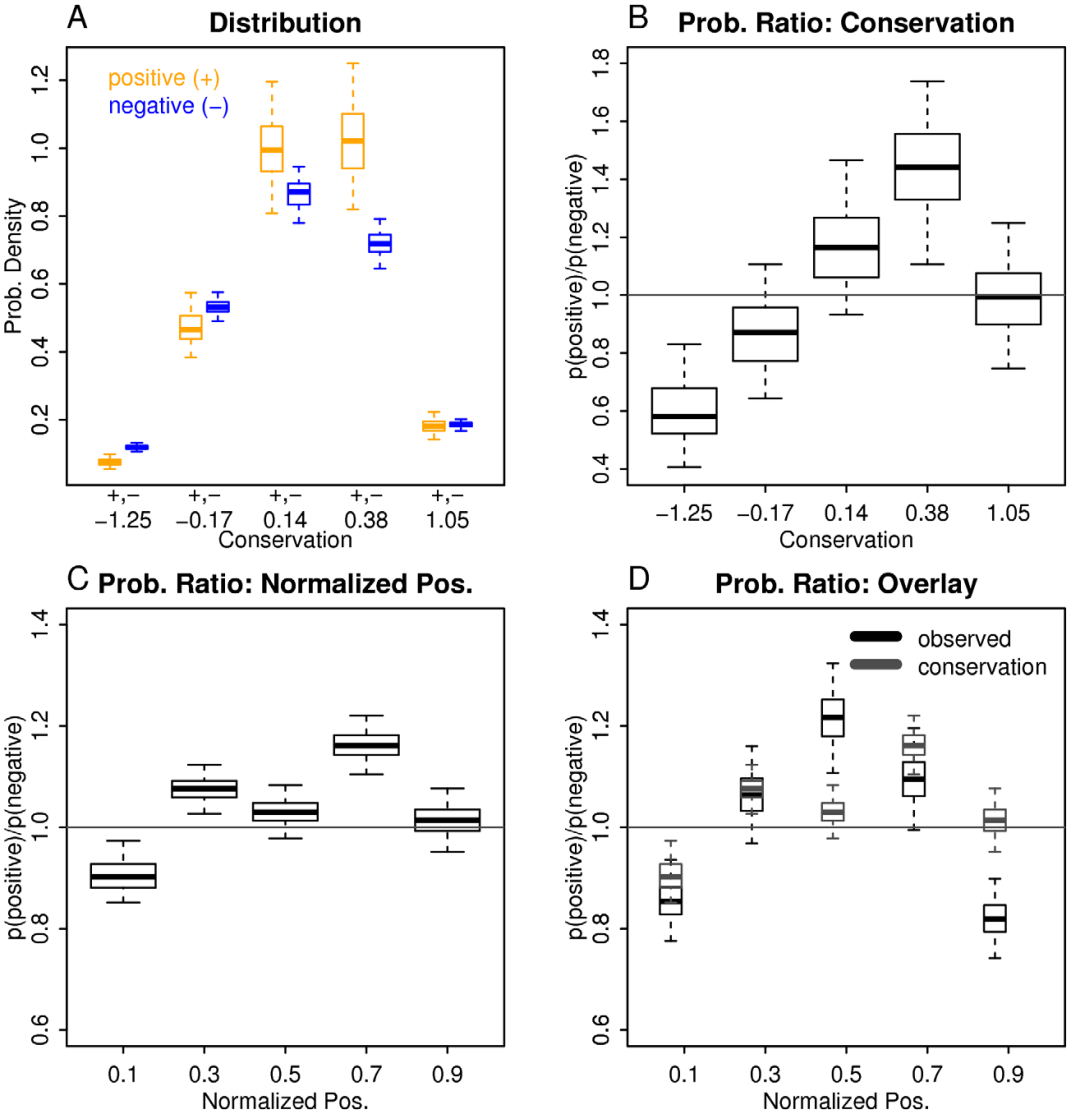
Estimating positional bias of epitopes from conservation data. (A) Bootstrap sampling of conservation scores for positive and negative peptides are shown as two boxplots placed next to each other. The bins used are of variable lengths to ensure a sufficient count in each bin. Each bin contains ∼20% of data points. Middle positions of bins are indicated on the x-axis. The difference between the means of the two conservation score distributions is statistically significant (Welch's t-test; one-sided; p-value = 5.9×10^−6^). (B) Estimating probability ratios from the conservation score distributions. This is simply taking a ratio of positive and negative peptide probabilities as a function of a conservation score. Confidence intervals are derived from bootstrap sampling. (C) Estimated probability ratios as a function of normalized position, using the mapping shown in the second panel. As input, distributions of means of conservation scores shown in Fig. 3 were used. (D) Positional bias curves derived from observed normalized positions (black) and conservation scores (gray).

To determine if sequence conservation alone can explain positional bias of epitopes, we first had to determine the relationship between conservation and immune recognition. In **Fig. 4A**, distributions of conservation scores for positives and negatives are shown. Positives tend to be more conserved than negatives (Welch's t-test; one-sided; p-value = 5.9×10^−8^). In **Fig. 4B**, a plot of probability ratio as a function of conservation score is shown. The plot is a result of calculating ratios of probability masses for positives and negatives shown in **Fig. 4A**. As shown in **Fig. 4B**, detecting epitopes is less likely as conservation decreases.

Next, we combined our estimates of conservation bias over the length of a protein with our estimate of correlation between conservation and immune recognition. Using conservation as a function of normalized position in **Fig. 3** as input, we estimated probability ratios as a function of normalized position (**Fig. 4C**). Its overlay with the probability ratios curve observed earlier (i.e. **Fig. 1C**) is shown in **Fig. 4D**. Overall, the probability ratios curve estimated only from the conservation data has a good agreement with the observed one (Pearson's correlation r = 0.57). In addition, for first, second and fourth bins, their confidence intervals between observed positional bias curve and that derived from conservation overlap. Thus, the observed positional bias in viral epitopes can be explained by the correlation between conservation and immune recognition alone, consistent with a minor contribution from premature translational termination and downstream initiation.

### Viral epitopes in the context of immunization with peptides display similar level of conservation as non-epitopes

There are two possible explanations for the observed correlation between immune recognition and conservation of peptides. One explanation is that the immune recognition machinery has evolved to preferably recognize epitopes that are conserved, as evidenced by an overlap of MHC binding motifs on conserved sequence regions found in [15]. An alternative explanation is that viral sequences are variable, and responses against epitopes from conserved regions could be higher in individuals that are exposed to multiple variants of a virus over time, as is expected for example in human influenza infections.

**Figure 5:**
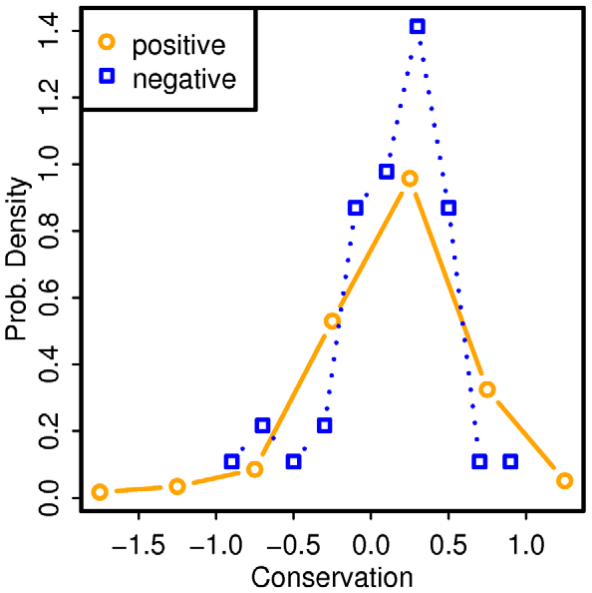
Distributions of conservation scores of peptides with positive and negative T-cell assay results for viruses in the context of immunization with peptides. The difference between the two distributions is not statistically significant (Welch's t-test; one-sided; p-value = 0.62).

To determine whether there is an intrinsic enhanced immune recognition for peptides that are conserved in viral species, we retrieved viral epitopes identified in the context of *peptide immunization*, rather than with viral infection. **Figure 5** shows distributions of conservation scores for positive and negative peptides, as was done earlier. The two distributions overlap, and their difference in means is not statistically significant (p-value = 0.62). Thus, we conclude that we cannot detect an intrinsically enhanced immune recognition for peptides that are conserved. This leads us to favor the hypothesis that the observed enhanced immune recognition of conserved viral peptides is due to extrinsic effects such as repeated priming of responses against conserved peptides due to heterologous exposure.

## Discussion

By leveraging a large set of experimentally determined epitopes from a wide range of viruses stored in the IEDB, we determined positional bias of epitopes in source antigens. The shape of positional bias curve (**Fig. 1**) shows significant under-representation of epitopes at N- and C- termini. This could be explained by near equal participation of downstream initiation and premature termination mechanisms in generating DRiPs as a major source of epitopes. Our findings, however, point to the conclusion that the central bias of viral epitopes reflects the combined effects of positional bias of epitope sequence conservation and induction of memory CD8+ T cells by exposure to heterologous boosts. There is a good correlation between positional bias curve estimated only from conservation data with that of the observed position bias curve of epitopes, so we cannot clearly detect (or clearly rule out) partially translated DRiPs transcripts as a major source of viral epitopes.

The connection between positional epitope biases with protein conservation is reasonable in the context of boosting effects associated with repeated vaccine administrations. The principle idea behind boosting is that those epitopes already exposed to the immune system tend to dominate in the following exposure [16]. This repeated exposure is a likely explanation of why MHC-I restricted viral epitopes tend to be more conserved than non-epitopes. Providing an additional support to this explanation, Kim et al. presented results showing positive correlation between epitope conservation and T-cell response frequency scores, which indicate how often individuals recognize a given peptide from a pathogen [17]. Presumably, this correlation is due to higher likelihood of more conserved epitopes being seen by greater number of individuals. In addition, as more immune epitope data are deposited at the IEDB, we expect to see differences in the degree of positional bias for RNA versus DNA viruses, because RNA viruses are more variable.

In addition to explanations discussed above, there are a number of recent ones that may be relevant. First, Calis et al. have reported correlations between G+C content and potential MHC-I binders [18], where low G+C content indicates pathogenicity. In our hands, we could not detect significant contribution of MHC-I binding affinity preferences to positional bias of epitopes, thereby making G+C content a less likely explanation. In explaining differential conservations between positive and negative peptides, it may be that this is due to viruses selectively mutating T-cell epitopes [19]–[21]. The idea is interesting and may be pursued in a future study. Our data set however takes protein variability as given and thus cannot be used to delineate its cause/effect relationships.

Other investigators have also reported epitope conservation for HIV [22], [23] and TB [24]. Our results extend their findings to a broad set of viruses, and suggest a possible connection between epitope conservation and boosting through analyses of epitopes determined in the context of immunization with peptides. It remains to be seen whether this boosting effect can be also observed for MHC class II restricted epitopes as well as in other immunological contexts.

Regarding the MHC-motif specific biases and conservation, it has been reported that predicted binding affinities of HLA molecules positively correlate with conserved regions of a wide range of viruses [25], which appears to contradict the results of absence of MHC-specific biases presented here. The absence of bias may be explained by the fact that the correlation between predicted binding affinity and protein conservation reported in [25] is very small to begin with (Spearman's rank correlation of at most ∼0.2), thereby dampening any MHC-specific biases that can be seen.

In conclusion, to better understand mechanistic details of antigen processing steps involving DRiPs, positional bias of MHC-I restricted viral T-cell epitopes was measured. Our findings indicate that there is indeed such bias in antigens, where epitopes at N- and C-termini are under-represented. Although mechanisms associated with translational errors such as downstream initiation and premature termination may contribute to observed positional bias, our data indicate that differential conservation spanning protein length is an alternative explanation.

## Methods

### Retrieving epitopes from the Immune Epitope Database

For each virus listed in **Table 1**, we retrieved MHC-I restricted T-cell epitopes from the Immune Epitope Database (IEDB) (http://www.iedb.org), which is the largest publicly available database of epitopes for infectious agents [8]. To retrieve an appropriate set of epitopes to examine the DRiPs hypothesis, there were a number of important considerations.

First, the query should retrieve epitopes derived from proteins newly expressed in a host cell, rather than epitopes recognized after peptide or protein immunization. Only for newly synthesized epitopes can defective ribosomal products skew the positional distribution of epitopes in antigens. To meet this requirement, we query the IEDB for epitopes identified using assays in which the ‘Immunogen Type’ is a whole ‘Organism’ (rather than an individual peptide or antigen). Second, we further limit the query to epitopes restricted by MHC class I molecules. Third, we limit the query to epitopes with ‘virus’ as the source organism.

We then grouped the epitopes retrieved with the query by viral species. As described below, we want to map all epitopes from one species to a single reference proteome. Therefore, we excluded all viruses for which we do not have reference proteomes available, resulting in a total of 93 different viruses.

### Mapping epitopes onto antigens of a complete proteome of an organism

To ensure consistent calculation of positional bias, we mapped all epitopes onto antigens from a single complete reference genome for each species based on sequence similarity rather than using the source antigens listed in the IEDB, which are those specified by the author mapping the epitope and are derived from different strains and are of divergent quality. For example, an author may have used truncated versions of an antigen, or epitopes may come from a polyprotein of Dengue virus, which later gets cleaved into individual products. Consequently, epitope positions can be made relative to the polyprotein or to final cleavage products.

To carry out the mapping, we used NCBI's BLAST with a default setting to search for presence of epitopes in antigens and to retrieve only those hits with exact matches in the reference genome. In addition, we required homology between the originally curated source antigen of the epitope and the antigen in the reference genome using BLAST searches with an E-value cutoff of 0.001, thereby ensuring meaningful mapping. Lastly, we required that there is only a single best match of the epitope in the reference genome to ensure that the position of the epitope in the antigen can be uniquely determined. We did not consider ties because of associated uncertainty in mapping.

### Calculating normalized positions of epitopes

To derive a measure of epitope position that is independent of protein length, a normalized position, *x*, is defined as follows:
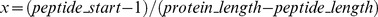
(1)In the equation, ‘*peptide_start*’ indicates the position of the first residue from the peptide mapped onto the protein sequence; ‘*protein_length*’ and ‘*peptide_length*’ are lengths of protein and peptide, respectively. The first residue of a protein has a position of 1, rather than 0. Our measure of normalized position, *x*, has the property that if a peptide contains the first residue of the protein (and thus comes from an N-terminal region), then its value is zero; if the peptide contains the last residue, then the value is 1.0. Consequently, if all regions of a protein are equally likely to contain epitopes, then one would obtain a uniform distribution of normalized positions in the range [0,1]. This property has been verified using randomly sampled peptide positions and uniformly sampled peptides from a set of proteins, followed by mapping the peptides onto the proteins with BLAST.

### Deriving probability ratios curve for measuring positional bias of epitopes

After mapping peptides with positive (i.e. epitopes) and negative T-cell assay outcomes onto their corresponding antigens, we calculated their normalized positions, x, as described in **Equation 1**. We then grouped the normalized positions into ‘positives’ and ‘negatives’, and binned using 5 bins of equal intervals covering a range [0,1], resulting in probability mass functions (PMFs): p(x|positive) and p(x|negative). The function p(x|positive) gives a probability of observing peptides at binned position *x*, given that only positives were considered.

To indicate positional bias, we calculated ratios of probability masses for positive and negative PMFs: p(x|positive)/p(x|negative). Absence of positional bias corresponds to a probability ratio of 1.0 for all bins. A probability ratio less than 1 indicates under-representation of epitopes while greater than 1 indicates over-representation.

### Estimating protein conservation at the residue-level

To determine whether differences in conservation over the normalized position in a protein contribute to positional bias of epitopes, we estimated conservations at the residue level for proteins from the viruses using Rate4site algorithm [26]. We chose the algorithm because it was identified as one of the highest performing methods in a recent benchmark to predict a known set of protein catalytic sites [27]. To estimate conservation, we used a protocol similar to one used by the ConSurf website [28]. Specifically, a multiple sequence alignment was generated for each protein by running a sequence of PSI-BLAST→CD-HIT→MUSCLE against the NCBI Non-Redundant database, using a set of Perl scripts retrieved from the ConSurf website. Running the conservation score estimation algorithm on the alignment returned residue position-specific conservation scores.

## Supporting Information

Figure S1 **Positional bias curves of epitopes after removing varying amounts of data.** For reference, the positional bias curve using all data is shown (‘all’). The curve labeled ‘v’ refers to exclusion of data from Vaccinia virus. The curve labeled ‘v+h’ refers to exclusion of data from vaccinia virus and Hepatitis C virus. Vaccinia virus had the largest amount of immune epitope data, followed by Hepatitis C virus.
(TIFF)

Figure S2 **Weighted combination of supertype-specific positional bias curves of predicted binders based on the frequencies of MHC restrictions observed for the known immune epitope data.**
(TIFF)

Figure S3 **Scatter plots of predicted binding affinities and their normalized positions for the known immune epitope data.** Top panels: normalized positions vs. predicted binding affinities. Bottom panels: bar plots where for each bar, a median of predicted binding affinities is shown. Red line indicates 500.0 nM cutoff. Orange line indicates a median of predicted binding affinities.
(TIFF)

Table S1 **Contingency table of number of peptides with positive and negative T-cell assay outcomes after binning based on their normalized positions.** The bins cover the range [0..1] and each bin has a fixed width of 0.2.
(XLS)

Table S2 **HLA supertypes and their member alleles used for peptide binding predictions.** Only those HLA alleles for which IEDB's SMM^PMBEC^ has 9-mer predictors available are shown.
(DOCX)
