# Supplementary figures and images for: Positional Bias of MHC Class I Restricted T-Cell Epitopes in Viral Antigens Is Likely due to a Bias in Conservation
Source: PLoS Comput Biol. 2013 Jan 24;9(1):e1002884. doi: 10.1371/journal.pcbi.1002884 (PMC3554532; doi:10.1371/journal.pcbi.1002884)

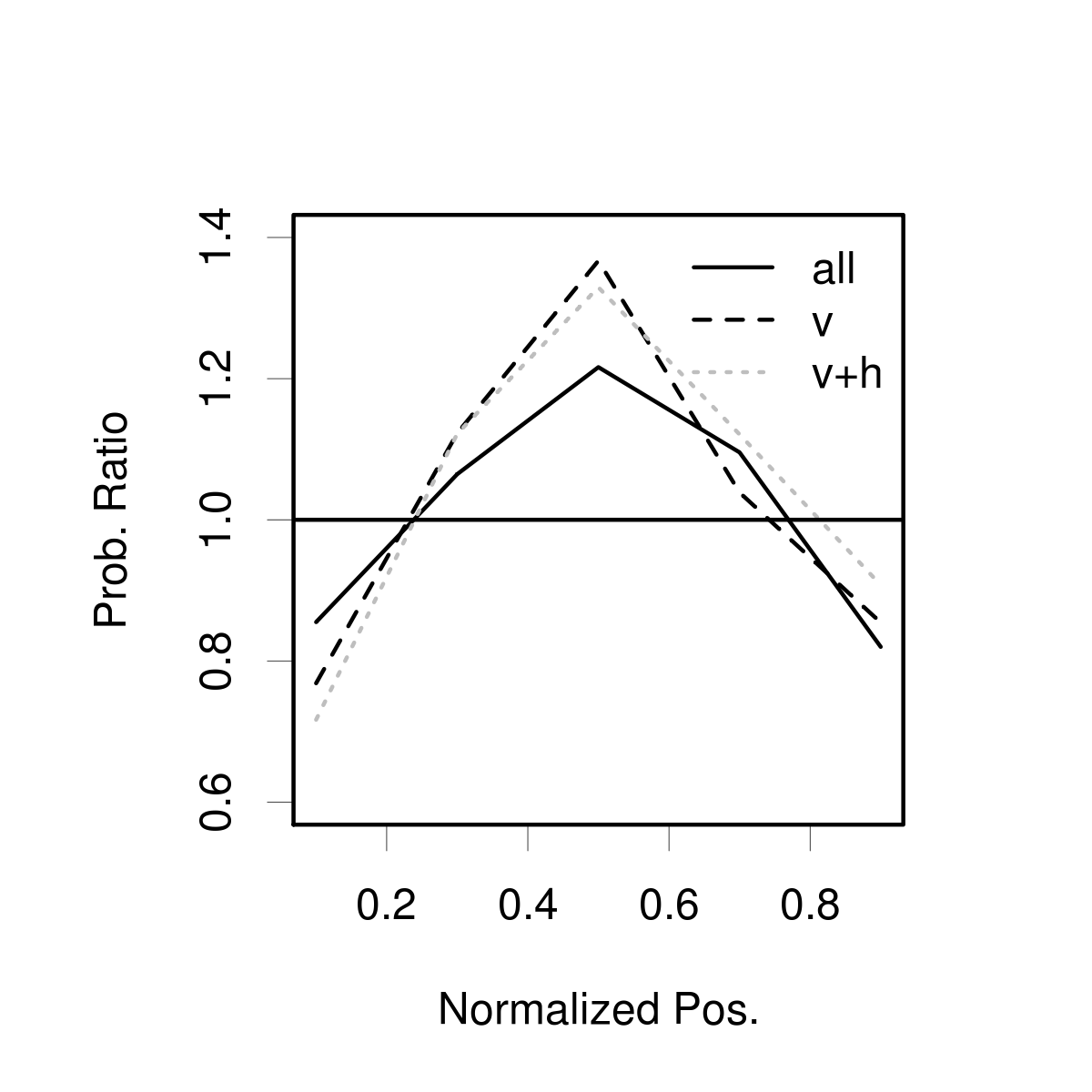

Supplement: Figure S1 — Positional bias curves of epitopes after removing varying amounts of data. For reference, the positional bias curve using all data is shown (‘all’). The curve labeled ‘v’ refers to exclusion of data from Vaccinia virus. The curve labeled ‘v+h’ refers to exclusion of data from vaccinia virus and Hepatitis C virus. Vaccinia virus had the largest amount of immune epitope data, followed by Hepatitis C virus. (TIFF) [file pcbi.1002884.s001.tiff]

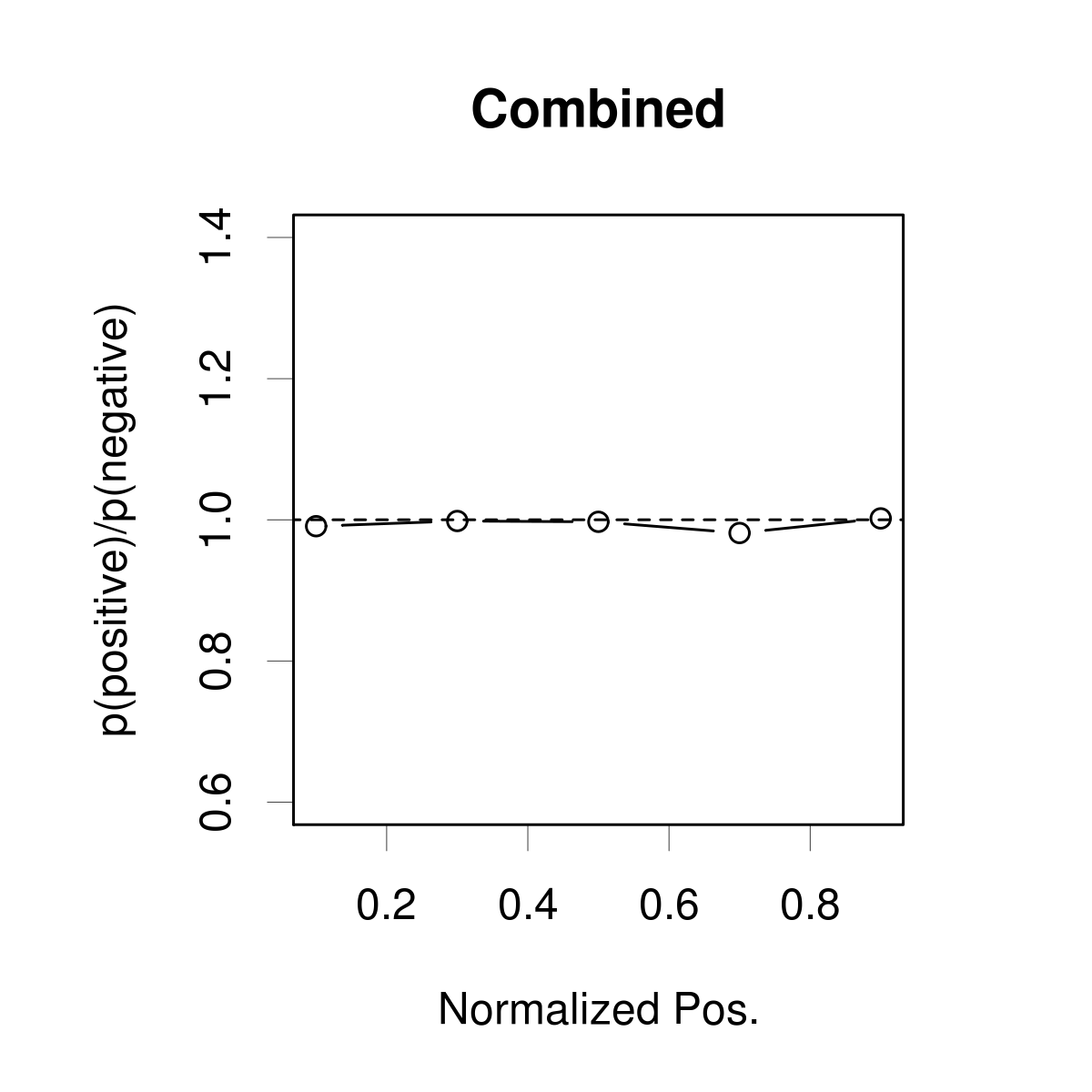

Supplement: Figure S2 — Weighted combination of supertype-specific positional bias curves of predicted binders based on the frequencies of MHC restrictions observed for the known immune epitope data. (TIFF) [file pcbi.1002884.s002.tiff]

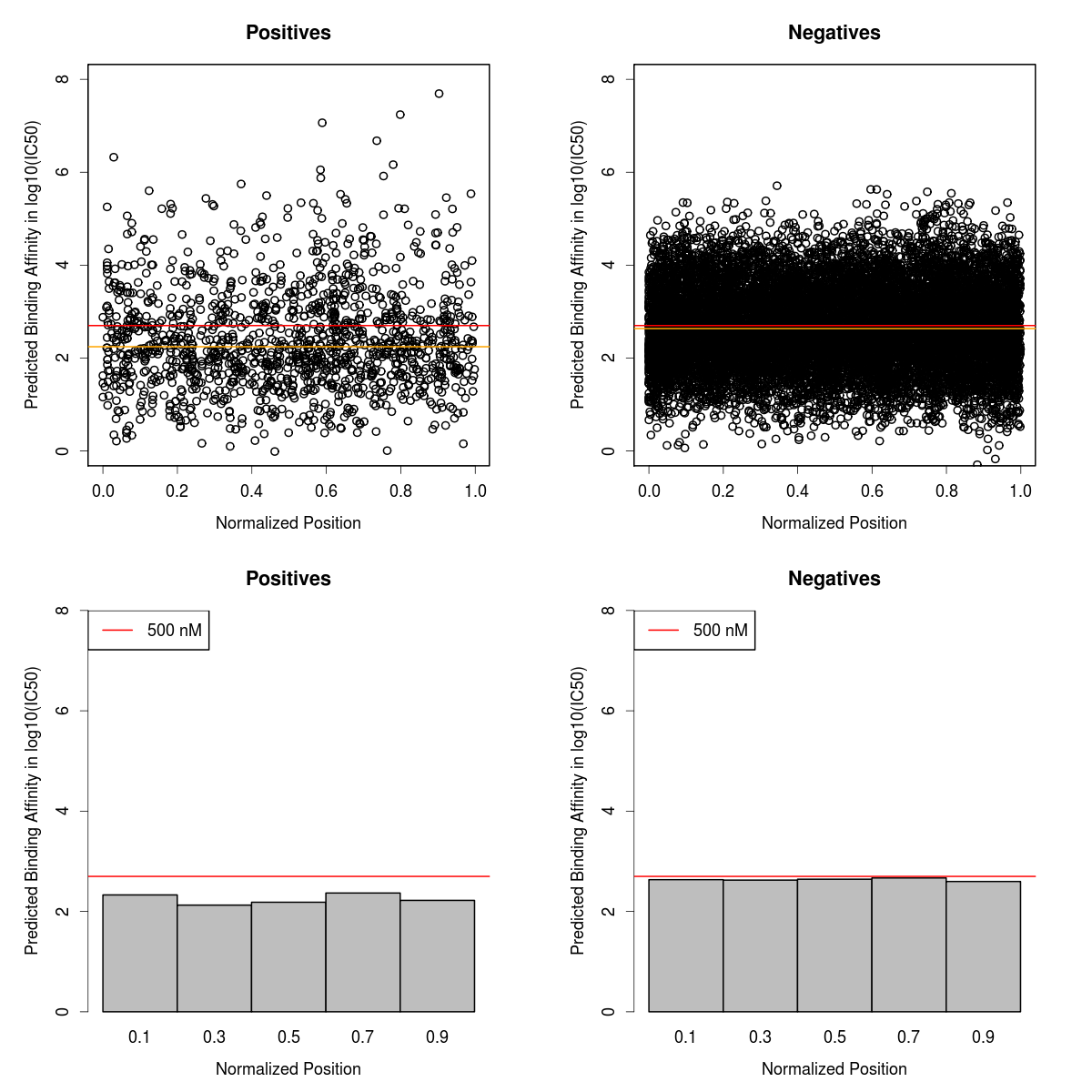

Supplement: Figure S3 — Scatter plots of predicted binding affinities and their normalized positions for the known immune epitope data. Top panels: normalized positions vs. predicted binding affinities. Bottom panels: bar plots where for each bar, a median of predicted binding affinities is shown. Red line indicates 500.0 nM cutoff. Orange line indicates a median of predicted binding affinities. (TIFF) [file pcbi.1002884.s003.tiff]
