# Supplementary material for: Positional Bias of MHC Class I Restricted T-Cell Epitopes in Viral Antigens Is Likely due to a Bias in Conservation
Source: PLoS Comput Biol. 2013 Jan 24;9(1):e1002884. doi: 10.1371/journal.pcbi.1002884 (PMC3554532; doi:10.1371/journal.pcbi.1002884)
Supplement: Table S2 — HLA supertypes and their member alleles used for peptide binding predictions. Only those HLA alleles for which IEDB's SMMPMBEC has 9-mer predictors available are shown. (DOCX) [file pcbi.1002884.s005.docx]

| **Supertype** | **Num. Alleles** | **Alleles** |
| --- | --- | --- |
| A01 | 8 | A*0101, A*2501, A*2601, A*2602, A*2603, A*3002, A*3201, A*8001 |
| A01A03 | 1 | A*3001 |
| A01A24 | 1 | A*2902 |
| A02 | 11 | A*0201, A*0202, A*0203, A*0206, A*0211, A*0212, A*0216, A*0219, A*0250, A*6802, A*6901 |
| A03 | 5 | A*0301, A*1101, A*3101, A*3301, A*6801 |
| A24 | 3 | A*2301, A*2402, A*2403 |
| B07 | 5 | B*0702, B*3501, B*5101, B*5301, B*5401 |
| B08 | 2 | B*0801, B*0802 |
| B27 | 7 | B*1503, B*1509, B*2705, B*3801, B*3901, B*4801, B*7301 |
| B44 | 6 | B*1801, B*4001, B*4002, B*4402, B*4403, B*4501 |
| B58 | 6 | B*1516, B*1517, B*5701, B*5702, B*5801, B*5802 |
| B62 | 5 | B*1501, B*1502, B*1512, B*1513, B*4601 |
